# On the Use of Oxygen in the Treatment of Anæmia, with a Case Treated by Transfusion of Blood

**Published:** 1896-09

**Authors:** J. Michell Clarke

**Affiliations:** Physician to the Bristol General Hospital, and Professor of Pathology and Morbid Anatomy, University College, Bristol


### ON THE USE OF OXYGEN IN THE TREATMENT OF ANÆMIA, WITH A CASE TREATED BY TRANSFUSION OF BLOOD.

BY

J. MICHELL CLARKE, M.A., M.D. Cantab, F.R.C.P. Lond.,

*Physician to the Bristol General Hospital, and Professor of Pathology and Morbid Anatomy, University College, Bristol.*

---

MY attention was first drawn to the treatment of anæmia by oxygen by Dr. Frederick Taylor's paper[1] on the subject. In the following observations the determinations of the corpuscles and hæmoglobin were made by Dr. Gowers's hæmocytometer and hæmoglobinometer. When there was any doubt as to their correctness they were made by two observers. In most of the cases blood films stained with eosin and methyl blue, and eosin and logwood, were examined for the condition of white corpuscles. The oxygen was administered by inhalation from an indiarubber bag, which was filled from a Brin's cylinder.

CASE 1.—Pernicious anæmia (advanced stage) treated by oxygen, arsenic, and transfusion of normal blood.

John B., labourer, aged 38, complained of extreme weakness and of pains in the back. The family history was good. The patient had suffered from dyspepsia, but had never had any bad illness.

The present illness began two years ago with a feeling of great weakness, followed shortly afterwards by a severe attack of diarrhœa, in which he rapidly lost flesh and strength, and was confined to bed for six weeks. The weakness has gradually increased, and he has latterly suffered from shortness of breath on the slightest exertion, and from swelling of the feet towards night. He has had great soreness of the mouth and tongue throughout the illness. He has had no cough. Once when suffering from piles he passed a small quantity of blood with the motions, but with that exception he has never had any hemorrhage from any source whatever. He has suffered from occasional attacks of diarrhœa for some years past.

On admission, May 9th, 1896, he was emaciated and extremely anæmic, the skin being of a pale primrose tint. He had diarrhœa, with the passage of pale, loose motions. Appetite bad. Temp. 98°. Tongue pale, with transverse lines. Pulse 96, regular, small, and compressible. Pupils large. Blood: flows with difficulty, of fairly good colour; hæmoglobin 31%; red corpuscles 810,000 per cubic millimetre; red corpuscles

[1] *Tr. Clin. Soc. Lond.*, 1895, xxviii. 47.

irregular in size and shape, many nucleated, they do not form *rouleaux*; white corpuscles not in excess, and normal in appearance (in stained specimens). The heart was not enlarged. A systolic soft (hæmic) murmur was heard all over the præcordial area—loudest over the pulmonary artery. Abdomen: there was a small quantity of ascitic fluid; neither liver nor spleen showed any enlargement; the skin of the abdomen was somewhat dark in tint (pigmented), and there were some rounded pigment spots over the back. Eyes: optic discs very pale, veins full; a small hemorrhage was seen to the inner side of the left disc, and there was also a retinal hemorrhage in the right eye. Urine: pale; contained no albumen or sugar; with the spectroscope it gave a distinct urobilin band; sp. gr. 1010. He was given hydronaphthol gr. v. every four hours, and ordered inhalations of oxygen.

May 12th.—A mixture containing subnitrate of bismuth and liq. opii sedativ. was substituted for the hydronaphthol.

May 28th.—The diarrhœa continued for a week and then ceased. He had inhaled 180 cubic feet of oxygen, and expressed himself as feeling rather better, but had to leave it off on several occasions on account of its increasing the soreness of his mouth. Blood: hæmoglobin 35%, red corpuscles 1,000,000 to c.m. Their appearance was as described above.

June 6th.—His tongue was covered with small blisters, and he complained much of soreness of the mouth, pharynx, and windpipe, increased by inhalation of oxygen, and on this account the inhalation of the gas was suspended.

June 9th.—During this week the temperature rose to 100°. The urine still showed a well-marked urobilin band. The administration of arsenic with strychnine was now begun. The diarrhœa returned and was troublesome for a week. Blood: red corpuscles 980,000 to c.m., hæmoglobin 30%.

June 25th.—He had been taking liq. arsenicalis steadily, until a dose of ♏ ix. three times a day had been reached. Latterly it had been given with liq. morphinæ, as it caused some irritation of the stomach. As the general condition remained about the same, and the man was very bad, it was thought advisable to try the injection of normal blood, as recommended by Dr. Brakenridge[1]. The operation of transfusion by the indirect method was carefully performed on June 26th, and 3 oz. of venous blood of normal composition as regards red corpuscles and hæmoglobin, together with 1½ oz. of sod. phosphate solution of sp. gr. 1031, were transfused. His blood was examined five hours after transfusion. The red corpuscles were 2,580,000 to c.m.; hæmoglobin 40%. No excess of white cells. The pulse was fuller and stronger.

June 27th.—Blood: red corpuscles 1,404,750; hæmoglobin 40%. Patient felt much better, and the pulse was better and stronger.

July 3rd.—Patient looked and felt better, but the feet and legs were more swollen, and the soreness of the mouth continued. Blood: red cells 1,440,000; hæmoglobin 40%. Microcytes and poikilocytes abundant. Also many large red corpuscles.

July 4th.—6 oz. of normal venous blood with 2 oz. of sod. phosphate solution were transfused at 1.30 p.m. Though the operation was done slowly with every precaution and, so far as could be seen, was correctly performed, the patient was very faint and bad for about an hour after it, the face being dusky and the forehead covered with cold perspiration. On examining the blood at 7.30 p.m., red corpuscles were 3,700,000. to c.m. At 9.30 p.m. he passed urine deep red in colour, containing a

[1] *Edinb. M. J.*, 1892–93, xxxviii. 409.

considerable quantity of albumen. It gave the spectroscopic bands of oxy-hæmoglobin. No corpuscles were seen under the microscope, but there was a deposit consisting of a quantity of small round bright yellow granules.

July 5th.—Urine, dark in colour, gave hæmoglobin bands, and contained a trace of albumen. Blood: red cells 2,120,000.

July 6th.—Urine contained no hæmoglobin. Blood: red corpuscles 1,540,000, hæmoglobin 40 %. The patient felt better for some days after the operation, with a stronger pulse. Temperature varied from normal to 100°.

July 15th.—As the patient was not so well, transfusion was again performed as on July 4th, the same amount of normal blood being injected into median basilic vein. As it was thought that the faintness on the former occasion might have been due to the injection of cocaine gr. ½ into the arm to produce local anæsthesia, this was omitted, and the operation performed very slowly and with great care. During the rest of the day the patient was in a very weak, almost collapsed, state, and passed three loose, very offensive motions, which however contained no blood. The urine remained clear on that day; but that passed next morning was dark brown in colour, contained albumen, a few hyaline casts, and a deposit of the same bright yellow granules as before. With the spectroscope the bands of oxy-hæmoglobin were visible. The urine became clear during the day, and next day contained no hæmoglobin. The temperature rose at 6 p.m. to 101·6°. Blood, examined ten hours after the operation, contained red corpuscles 1,650,000, hæmoglobin 40%.

One week later the patient became much worse, and died on July 26th. It may be said briefly that the *post-mortem* appearances were those associated with pernicious anæmia. There was a moderate degree of fatty degeneration of the heart muscle; the liver and kidney gave reactions for iron; and the marrow in the tibia and sternum was red and semi-fluid. The spleen was not enlarged. There was a small quantity of fluid in the pleural and abdominal cavities, which was of a bright yellow colour.

With regard to treatment the oxygen inhalations, though carried out regularly at first, seemed to give little benefit, and they had to be discontinued on account of the soreness of the mouth and trachea, and of the feeling of extreme distension they produced. The administration of arsenic was attended with no benefit. On each occasion after transfusion of blood the condition of the patient's blood, as regards number of red cells and percentage of hæmoglobin, was temporarily improved, and this was most marked on the second occasion. It was noticeable that the percentage of hæmoglobin was raised after the first transfusion, and remained about the same level afterwards. The patient also felt better after them. The improvement was however merely temporary, the condition of the blood steadily reverting to its former state, and in spite of due care in the operation the last two transfusions were attended with the serious symptoms above given. The disease was however far advanced on admission, and possibly treatment was undertaken too late.

CASE 2.—Case of profound anæmia in which recovery occurred under the use of oxygen. Clinically this case appeared to be one of pernicious anæmia, but there was also present a slight enlargement of the spleen.

Emily T., aged 54. Family history good. The patient had always been delicate in health, and suffered from frequent attacks of sick-headache, but was active and energetic, and had never had any bad illness. She complained of weakness, palpitation, of attacks of sickness, and of constant headache. Eighteen months ago she had an attack of jaundice, and the whole illness had lasted about two years, being attributed by her to grief and anxiety. During the previous winter she was laid up in bed for three or four months with a bad cold and weakness. Since that time she had suffered from coldness and numbness of the extremities. During the six weeks previous to admission into the Bristol General Hospital, on Sept. 17, 1895, she had become so weak that she could only walk with the greatest difficulty. Her breath was very short, and there was some œdema of the feet at night. She had never suffered from hemorrhages of any kind.

On admission the patient was well nourished, but very anæmic, the skin having a lemon-coloured tinge. The pulse was 84, soft and compressible; respirations 22; temperature 99°. The lungs were healthy. The heart was slightly enlarged; a soft systolic murmur was audible all over the præcordial region, and hæmic murmurs were heard at the root of the neck. The liver was not enlarged. The spleen was slightly enlarged, its tip being plainly felt below the ribs. The skin of the abdomen was slightly pigmented and dark in colour. Urine: clear, contained no albumen or sugar, gave a well-marked indigo reaction, sp. gr. 1010; average daily amount, 45 oz.; no urobilin band was seen with the spectroscope; urea 1.5%. Optic discs and retinæ were pale, but no hemorrhages were seen. Blood: red cells 2,200,000 to c.m.; many poikilocytes, macrocytes, and microcytes, and a few nucleated corpuscles; no excess of white corpuscles; hæmoglobin 55%.

For the first month she was treated with increasing doses of arsenic. She suffered at times from troublesome attacks of diarrhœa, with the passage of loose and offensive motions, for which she took a mixture containing bismuth subnitrate and salol. Towards the end of this time she also took a preparation of marrow. Her temperature remained about normal, except on two days when it rose to 100°. At the end of the month she showed no improvement, excepting a slight increase of appetite. Examination of the blood now showed: red corpuscles 2,090,000 to c.m., hæmoglobin 52%.

Oct. 17.—Inhalations of oxygen begun. After a week's administration of oxygen she felt decidedly better and stronger; she felt brighter immediately after the inhalation, often falling into a sleep and awaking refreshed.

Nov. 4th.—She had inhaled 40 c. ft. of oxygen. Blood: red corpuscles 2,390,000, hæmoglobin 55%; characters of corpuscles the same as on admission. Arsenic was now discontinued on account of sickness.

Nov. 30th.—Up to this date she had inhaled five cylinders of oxygen, each containing 40 c. ft., using them at the rate of one cylinder in five days. Blood: red corpuscles 2,940,000 to c.m., hæmoglobin 68%. It was noticeable that the number of corpuscles began to increase before the percentage of hæmoglobin was raised. She complained of a little soreness of the mouth after inhaling oxygen.

Dec. 18th.—The administration of oxygen was continued in the same quantity. Blood: hæmoglobin 78%, red corpuscles 3,960,000; corpuscles mostly normal in size and shape, but a few poikilocytes present in each field; the blood flowed more readily from the finger.

Jan. 4th.—She had greatly improved in strength, the attacks of headache and sickness had ceased, and she suffered only very slightly from flatulence. It was doubtful whether the tip of the spleen could be felt or not. She left the hospital and attended as an out-patient, again taking small doses of arsenic.

She continued to attend as an out-patient, taking arsenic regularly.

July 23rd.—She looked well, was able to get about and do her housework, and had gained flesh. Skin of abdomen was now deeply pigmented, especially on left side. No enlargement of the spleen could now be detected. Heart: no murmur at apex; ? faint systolic over pulmonary area. Blood: hæmoglobin 70%; red corpuscles form *rouleaux* and are of normal appearance, 4,000,000 to c.m.; no excess of white cells.

CASE 3.—Profound anæmia, probably due to repeated small attacks of epistaxis, relieved by oxygen inhalations.

Geo. W., aged 43, an agricultural labourer, his work entailing a good deal of exposure to cold and wet. Nothing of importance in family history. He had always enjoyed good health except for an attack of small-pox three years ago, and of influenza four years ago. Ever since a child he has been subject to occasional attacks of epistaxis, sometimes with the loss of a considerable amount of blood; he has not especially suffered in this way lately. He often suffers from heavy colds, which settle on his chest, and the epistaxis is then worse. No bleeding from any other source. He had never suffered from piles, or had any venereal disease.

For the last two or three months the patient has suffered from pain and sensation of weight in his stomach immediately after taking food; also from shortness of breath, and latterly from palpitation of the heart after exertion. His feet swell towards night.

Dec. 14th, 1895.—On admission he looked profoundly anæmic; there were some stigmata and bright red rounded papules on cheeks. Pulse 70, sudden, short wave, not easily compressible. Temperature 98°. Appetite good. Bowels regular. Tongue: small, pale, moist. He was well-nourished, and the skin of a faint lemon tint. Chest normal, except that there is a little deficient resonance and weakness of breath-sounds over left side. Heart: apex-beat 5th space 1 in. outside nipple. There was a little deficient resonance over the 1st piece of the sternum. A systolic murmur was audible over the whole præcordial area—loudest in the 2nd left intercostal space, and at the apex. The aortic 2nd sound was accentuated. Liver and spleen of normal size to percussion, latter not felt. Urine: clear, acid, no deposit; sp. gr. 1010; no albumen or sugar. Nails indented, transversely furrowed and cracked. Blood: corpuscles 3,100,000 to c.m., hæmoglobin 31%; red corpuscles irregular in size and shape, and there were a few large and very many small corpuscles. The number of leucocytes was slightly increased. The blood was rather watery and flowed with difficulty. Optic discs pale. No retinal hemorrhages.

Treatment until January 1st was by arsenic in gradually increasing doses, with ferri et ammon. cit. gr. x. three times a day. Little or no improvement resulted, the condition of the blood and the general symptoms remaining the same. The temperature was not above normal, with a tendency to fall to 98°. The faint icteric tinge of the skin remained present.

Jan. 1st.—The administration of oxygen was begun, and by the 14th he had inhaled 120 cubic feet of the gas. During this fortnight he had a mild attack of bronchitis and two attacks of epistaxis. Examination of the blood on Jan. 14th gave hæmoglobin 34%; red corpuscles 3,800,000 to c.m. They formed *rouleaux* and poikilocytes were fewer in number.

During the following three weeks he inhaled 270 cubic feet of oxygen. His condition steadily improved, although he had several attacks of epistaxis. The loss of blood in each attack was, however, never considerable, and was controlled by the use of a tannic acid snuff.

Feb. 4th.—The red corpuscles were 4,200,000 to c.m., and the hæmoglobin 45%. There were still present small and irregularly shaped corpuscles, but taken as a whole the corpuscles were much more regular in size and shape than on admission.

Feb. 14th.—As the rise in the percentage of hæmoglobin had not kept pace with that in the number of the red corpuscles, the patient was given three grains of the exsiccated sulphate of iron three times a day. The oxygen inhalations were continued.

The subsequent course of the case was one of steady improvement; the epistaxis ceased, and on March 7th the hæmoglobin had risen to 65% and the red corpuscles to 4,300,000 to c.m.; the latter were of good shape and formed *rouleaux*.

He left the hospital on March 9th, feeling and looking well, and came up as an out-patient on April 20th, reporting himself as well and able to do his work. On examination of the blood the red corpuscles were 4,300,000 to c.m. and hæmoglobin 70%.

Here the patient's aspect and the microscopic appearances of the red corpuscles were indistinguishable from those of pernicious anæmia; but on the other hand the reduction in the percentage of hæmoglobin was not proportional to the destruction of red corpuscles, the blood decimal on admission being 0.5; in this respect, therefore, the condition of the blood corresponded to that found in chlorosis. Moreover, there were no retinal hemorrhages, nor was there a urobilin band in the urine. The source of the anæmia appeared to be the repeated small losses of blood from epistaxis, for which no local cause could be found in the nose. The beneficial effect of oxygen was marked in this case, and, as in the second one, it was noted that a rise in the percentage of red corpuscles occurred without a proportionate increase in hæmoglobin, whilst a more rapid improvement in the latter respect took place on giving iron in addition to oxygen.

Case 4.—Case of anæmia following *post-partum* hemorrhage.

The patient, aged 33, had enjoyed good health until her confinement six weeks previously, which was followed by excessive hemorrhage. On admission she was extremely weak, and presented the appearance of profound anæmia. She answered questions very slowly, and had become deaf during the present illness. A systolic murmur was heard over the area of the pulmonary artery, otherwise the thoracic and abdominal organs were normal on examination. The temperature was normal. Blood: hæmoglobin 15%; red corpuscles 780,000 to c.m., they formed *rouleaux* imperfectly, looked pale, and there were numerous microcytes and poikilocytes. Blood decimal 0.9.

She was given a ferruginous mixture, and in addition inhaled about 34 gallons of oxygen daily. She experienced great relief to her symptoms from the inhalations of oxygen, and made a rapid recovery, being fit to leave the hospital just under six weeks from admission, examination of the blood then showing red corpuscles 4,110,000 to c.m., and normal in size and shape; hæmoglobin 79%.

Judging from the severe degree of anæmia present on admission, recovery appeared to take place more rapidly than it would have done under treatment with iron and rest only.

These cases seem to show that considerable benefit may be derived from the use of oxygen in the treatment of cases of profound anæmia, and that under its employment recovery may be more speedy and complete. In the milder forms of anæmia and chlorosis it will hardly be necessary, and in the severe cases, as well as in pernicious anæmia, I think that its use will be rather as an adjuvant to the established remedies, iron and arsenic, than as a substitute for them. In the 2nd and 3rd cases described above, improvement took place under oxygen, when arsenic in the former, and arsenic and iron in the latter, had failed at first to do good. The only drawback noticed in its administration was that it caused in two of the patients a painful soreness of the mouth and throat and a feeling of distension.

